# Process utilities for topical treatment in atopic dermatitis

**DOI:** 10.1007/s11136-019-02174-0

**Published:** 2019-04-03

**Authors:** Jenny Retzler, Adam Smith, Matthew Reaney, Raj Rout, Richard Hudson

**Affiliations:** 1 York Health Economics Consortium, University of York, York, UK; 2 Department of Psychology, University of Huddersfield, Huddersfield, UK; 3 Sanofi Genzyme UK and Ireland, One Onslow Street, Guildford, Surrey, GU1 4SY UK

**Keywords:** Utility elicitation, Time trade-off, Atopic dermatitis, Process utility, Topical treatment

## Abstract

**Purpose:**

Management of atopic dermatitis (AD) typically requires application of topical treatments, often multiple times a day. The cosmetic properties and burdensome application of these treatments can be detrimental to quality of life (QoL). Patients who achieve good disease control through use of systemic therapies may reduce the frequency and amount of topical applications, improving QoL. This study aimed to quantify the utility and disutility for topical AD treatment processes.

**Methods:**

Seven vignettes describing different skincare regimens for people with moderate-to-severe AD were developed with input from healthcare professionals. 484 respondents from the general population completed time trade-off items for each vignette. Utility values for each regimen, and disutilities associated with the impact of changes to skincare regimens, were calculated. Analysis of variance assessed differences between skincare regimens.

**Results:**

As skincare regimens increased in intensity (0.7968 for the most intense; 0.9999 for the least), utility values decreased. There were no statistically significant differences between skincare regimens followed by patients with good disease control (0.9862 to 0.9999); however, when compared to those involving topical corticosteroids and emollient combinations (0.7968 to 0.8835), significant differences were observed (*p *< 0.001). The largest disutilities (0.1521 to 0.1705) were between skincare regimens describing the use of topical corticosteroids plus emollient and those followed by patients with good disease control.

**Conclusions:**

The application of topical treatments has a detrimental effect on QoL, which increases with the duration and frequency of applications. Further research is needed to investigate how health and process utilities interact and both can be integrated into medical decision-making.

**Electronic supplementary material:**

The online version of this article (10.1007/s11136-019-02174-0) contains supplementary material, which is available to authorized users.

## Introduction

Atopic dermatitis (AD) is an immune-mediated chronic inflammatory skin disease, characterised by persistent and intense itching and redness, and rough, flaking, dry skin with oozing, bleeding and cracking. The estimated global prevalence for AD in adults ranges from 2 to 5% [1, 2] and is associated with significant disease burden. For patients with moderate-to-severe AD, disease exacerbation occurs for 4 to 6 months of the year [3], and AD impacts on sleep, physical, emotional and social functioning, and has been associated with depression, all of which detrimentally influence quality of life [4–6].

### AD management and the quality of life associated with the impact of treatment processes

The mainstay of AD management is topical therapy. Moisturisers and emollients are recommended for all patients to improve skin-barrier function and reduce water loss, and topical corticosteroids (TCS) or calcineurin inhibitors are indicated when non-pharmacological interventions alone are insufficient [7]. However, the application of topical treatments is burdensome, and patients often complain of the impact of unpleasant cosmetic properties. Surprisingly, little work has systematically appraised the demands and burden of topical treatment as perceived by AD patients, and much of it would be considered ‘grey literature’. Qualitative syntheses of perceptions of patients, such as those on patient support websites (e.g. Health Talk [8]), indicate that topical treatments can cause embarrassment in social situations, and patients complain of the ‘hard work’ involved with applying topical treatments, and that they miss out on activities in order to stick to treatment routines. Surveys have taken a more quantitative approach. For instance, an Allergy UK survey reported that 86% respondents found that AD management impacted their daily activities, while 10% spent more than 2 h each day on symptom management [9]. A survey published in a peer-reviewed journal described similar results; 60% patients with severe AD were unhappy that they were restricted in clothing choice, 82% were concerned about their appearance, and 86% had to avoid at least one type of everyday activity when they had an AD flare [3]. There are also reports in the paediatric AD literature of how disease management significantly affects aspects of family lifestyle and activities [4]. For instance, the daily time required to treat a child with severe AD can range from 1 to 3 h per day on average [10, 11].

Across various diseases, factors associated with treatment administration have been shown to affect patient perceptions of treatments beyond safety and efficacy considerations [12], and this may be linked to the detrimental impact on QoL that treatment administration can have. Whilst, to our knowledge, this has not been explored in AD, a review of treatment processes in psoriatic patients investigated preferences for different types of treatment administration [13]. Patients preferred topical creams and ointments over lotions and gels, while aspects such as administration frequency for injected and oral medication were also important. Similarly, evidence indicates that non-adherence in psoriasis can be associated with process aspects, including the application time and unpleasant cosmetic characteristics (“sticky sensation”) of topical treatments [14]. If treatment processes reduce adherence, they may consequently reduce therapeutic benefit. Moreover, dissatisfaction with treatment has itself been shown to negatively impact QoL in psoriatic patients on topical treatments [15].

In moderate-to-severe AD refractory to topical treatments, systemic immunosuppressants are used both on- and off-label. Oral steroids and phototherapy may be also used in the short term to manage exacerbations [16, 17]. Where these systemic therapies are effective, it is likely that patients will typically reduce the frequency and volume of burdensome topical applications if they are able to maintain good disease control, which may, in turn, reduce some of the QoL burden.

### Quantifying the impact of treatment benefits

Economic evaluations of new treatments use the concept of health utility to incorporate treatment benefit into analyses. Health utilities, which measure the value a population places on the impact of a disease on health, can be captured using indirect utility questionnaires such as the EQ-5D and SF-6D [18, 19] in patient populations, or directly using elicitation methods such as the time trade-off (TTO) procedure in patients (rating their own health) or the general population (rating a hypothetical health state described in a vignette) [20]. The TTO procedure asks individuals to trade a number of years of life in a given health state against fewer years in perfect health. This approach may also be used to estimate the value a population places on treatment processes (such as the application of topical therapies), thereby generating a ‘process’ utility value quantifying non-health benefits of new treatments which could be used in medical decision-making.

Studies have demonstrated a range of process utilities associated with dosing strategies, ease of use and route of administration in various indications (see Brennan and Dixon [21] for a systematic review), but none have quantified process utility in relation to the management of AD. Given the reported impact of topical treatment in AD on the individual patient, it is of importance to capture the utility or disutility associated with such regimens, particularly in the context of the potential for new therapies to alter the treatment management burden. The aim of this study was, therefore, to estimate the utility associated with different topical skincare regimens that patients may follow to manage of moderate-to-severe AD according to the UK general population.

## Methods

### Skincare regimens

Telephone interviews were conducted with three nurses who specialised in AD care to establish (i) the typical requirements for the application of emollient and TCS, (ii) the impact of emollient and TCS on patients’ daily life, and (iii) the ways in which emollient and TCS use changes in patients who achieve good disease control with the use of systemic therapies.

Following collation of the interviews, 7 skincare regimens were established for the utility elicitation process (Table 1). These skincare regimens represent those that AD patients using traditional TCS + emollient products may be required to follow (#1 to #3), and different plausible scenarios of skincare regimens that may be followed by patients with good disease control resulting from use of systemic therapies (#4 to #7).

**Table 1:** Skincare regimens for utility elicitation

No.	Skincare regimen	Scenario
1	Steroid twice daily and emollient four times daily	Skincare regimen advised for severe AD patients
2	Steroid twice daily and emollient twice daily	Typical skincare regimen followed by severe AD patients unwilling/unable to follow recommended regimen, therefore limiting emollient applications
3	Steroid once daily and emollient twice daily	Advised skincare regimen for moderate AD patients OR Regimen followed by severe AD patients unwilling/unable to follow the recommended regime, therefore limiting both steroid and emollient applications
4	Light emollient twice daily	Skincare regimen advised for patients with good disease control
5	Light emollient once daily	Possible skincare regimen followed by patients with good disease control
6	Light emollient once every other day	Possible skincare regimen followed by patients with very good disease control
7	Light emollient on occasion, as needed	Possible skincare regimen followed by patients with very good disease control

### Elicitation methods

A TTO task was developed for the study. Respondents were asked to imagine they were living with AD. Next, they were presented with each of the seven skincare regimens one-by-one, in a random order, and asked to imagine they have 10 years left to live, throughout which they will have to follow the skincare regimen described. They were then given the option of living for a shorter time (i.e. trading-in some of their remaining life) in order to no longer need to follow the skincare routine described above (no resolution of AD itself was offered). If they decided that they would give up some time, they were asked to indicate the maximum length of time they would give up. This length of time traded-off corresponded to the process utility as follows:

1- (Length of time traded-off/10)

### Survey design

A survey was developed using the Qualtrics online survey platform [22]. Part one recorded demographic information (e.g. age, gender and ethnicity) about the respondent, as well current or previous use of TCS to treat skin conditions. Part two presented each of the 7 skincare regimens in turn (randomised order) with the TTO items (see Supplement B for a copy of the full survey with all TTO items). Briefly, each skincare regimen description described the types of products required, the frequency and duration of applications, and ways in which these applications may affect daily life.

The survey was finalised after two rounds of pilot testing to assess the usability and functionality of the survey. Feedback from a total of 16 respondents of different educational backgrounds and ages was used to inform revisions to ensure ease of comprehension and usability.

### Procedure

A general population sample was recruited via an email invitation by Qualtrics [22]. Online panel respondents received electronic points in exchange for taking part in the online survey. Consenting respondents were pre-screened via the demographic questions to ensure that only those who met eligibility criteria completed the full survey. The sample was selected to be broadly representative of the adult general population in the UK. Respondents were eligible to participate provided they were a resident of the UK over the age of 18 years. However, in order to achieve a representative sample, quotas were set based on the socio-demographic variables (Table 2). Recruitment was planned to continue until a total of 500 complete responses were received. No data from incomplete responses were saved, thus the final dataset included only complete responses. Presentation of the skincare regimens was randomised with even presentation using the in-built algorithm on the Qualtrics platform.

**Table 2:** Target and actual sample characteristics

Characteristic	Target sample (%)	Actual sample
Gender^a^		
Male	49	229 (47.3%)
Female	51	255 (52.7%)
Age^b^		
18–24	11	55 (11.4%)
25–34	17	85 (17.6%)
35–44	16	80 (16.5%)
45–54	18	90 (18.6%)
55–64	14	70 (14.5%)
65 and over	23	104 (21.5%)
Ethnicity^c^		
White or White British	87	435 (89.9%)
Asian or Asian British	7	22 (4.5%)
Black or Black British	3	13 (2.7%)
Mixed or Multiple	2	9 (1.9%)
Other	1	5 (1.0%)
Previous use of steroid creams		
Yes	–	214 (44.2%)
No	–	270 (55.8%)
Country of habitation		
England	–	413 (85.3%)
Scotland	–	40 (9.3%)
Wales	–	24 (5.0%)
Northern Ireland	–	7 (1.4%)
Device		
Smartphone	–	14 (2.9%)
Laptop	–	250 (51.7%)
Computer	–	137 (28.3%)
Tablet	–	83 (17.1%)

^a^Eurostat, 2015 data. Proportion of males to females calculated from total population data

^b^Eurostat, 2015 data, age-group data summed into appropriate categories. To calculate data for the 18–24 category, 40% of 15–19 age group total was added to the 20–24 total. Proportion of the total adult population (18+) for each category was then calculated

^c^Census 2011 data calculated from total population data

### Statistical analysis

Data were exported into SPSS (version 24.0) for analysis. Descriptive statistics detailing the socio-demographic data for the sample were generated.

Outliers (1.5 interquartile ranges above the upper quartile or below the lower quartile [23]) for each skincare regimen were filtered from relevant analyses. Mean process utility values associated with each of the skincare regimens were calculated for each respondent and aggregated for each regimen (Table 3). Process disutilities, which reflect the decrement in utility due to a particular difference between two skincare regimens that are identical in all aspects aside from burdensomeness [e.g. two TCS applications vs. one TCS application in conjunction with emollient use twice daily (skincare regimen #3 vs #2)], were calculated only between regimens with theoretically relevant differences defined a priori (#1 vs #4; #1 vs. #7; #1 vs. #2; #2 vs. #3; #2 vs. #4; #3 vs. #4; #4 vs. #5; and #5 vs. #6; see Table 4). Disutility values were calculated both from respondent-level data (including only respondents with non-outlying responses for both of the two skincare regimens in question) before aggregation, as well as from the grand means [the difference between aggregated mean process utilities for the two skincare regimens in question (Table 3), resulting in different numbers of respondents contributing to each part of the calculation].

**Table 3:** Average utility values for each skincare regimen

No.	Skincare regimen	*N*	Mean (SD)
1	Steroid twice daily and emollient four times daily	473	0.7968 (0.2159)
2	Steroid twice daily and emollient twice daily	466	0.8471 (0.1744)
3	Steroid once daily and emollient twice daily	446	0.8835 (0.1469)
4	Light emollient twice daily	404	0.9862 (0.0340)
5	Light emollient once daily	396	0.9906 (0.0267)
6	Light emollient once every other day	370	0.9997 (0.0021)
7	Light emollient on occasion, as needed	371	0.9999 (0.0012)

**Table 4:** Average disutility values associated with changes in skincare regimens

Nos.	Change in skincare regimen	Generated at the respondent level		Generated from the grand means observed in Table 4
		*N*	Mean (SD)	Mean
1 versus 4	Steroid + emollient as advised vs good disease control as advised	398	− 0.1521 (0.1871)	− 0.1894
1 versus 7	Steroid + emollient as advised vs best case scenario with very good disease control	364	− 0.1705 (0.1907)	− 0.2031
1 versus 2	2 additional applications of emollient in patients using steroid + emollient	461	− 0.0436 (0.1504)	− 0.0503
2 versus 3	1 additional application of steroid in patients using steroid + emollient	445	− 0.0214 (0.1151)	− 0.0364
2 versus 4	2 applications of steroid, plus heavier emollient in patients applying 2 applications of emollient	398	− 0.1081 (0.1496)	− 0.1391
3 versus 4	1 application of steroid, plus heavier emollient in patients applying 2 applications of emollient	398	− 0.0822 (0.1260)	− 0.1027
4 versus 5	1 additional application of light emollient in patients with good disease control	373	− 0.0039 (0.0291)	− 0.0044
5 versus 6	Daily versus bi-daily application of light emollient in patients with good disease control	346	− 0.0049 (0.0196)	− 0.0002

A mixed ANOVA including respondents with non-outlying data for all skincare regimens (*n* = 304; 62.8% of total sample) was used to assess within-respondent differences between skincare regimens, and between-respondents differences in previous TCS use (yes, no) and the device of completion (smartphone, laptop, tablet, computer, etc). Assumptions of sphericity and homogeneity of variance were assessed using Mauchly’s test and Levene’s test, respectively. Sensitivity analyses involving alternative outlier removal methods were conducted to assess the impact of the outlier removal method (Supplement A).

## Results

### Sample

A total of 484 respondents completed the survey. Due to recruitment challenges, the target quotas for the categories of male, 65 and over and Asian or Asian British were not quite met, resulting in a slight overrepresentation of White and White British ethnicities (Table 2). As such, a decision was taken to stop sampling at 484 respondents to achieve a sample that was broadly representative of the UK population in terms of gender, age, and ethnicity. Just under half (44%) of respondents reported having used TCS to treat skin conditions.

### Utilities

Average utilities, displayed in Table 3, were consistent with expectations, whereby more intensive skincare regimens were rated as having the lowest utilities. There was very little difference in utility between skincare regimens likely to be followed by patients with good disease control (#4 to #7), all of which had utility values close to perfect health (0.9862 to 0.9999), while values were lower for skincare regimens likely to be followed by patients using TCS and emollient treatments (#1 to #3; 0.7968 to 0.8835).

The assumption of sphericity was violated, thus Greenhouse–Geisser degrees of freedom were reported for within-respondents analyses. While the assumption of homogeneity of variance was violated for some variables, no between-respondents factors were significant, and as violations of this type increase the risk for Type 1 errors, no adjustments to the degrees of freedom were made.

There was no main effect of either of the between-respondents variables; utility values differed neither between those who had or those who had not used TCS to treat skin conditions previously [*F*(1, 296) = 0, *p *= 0.999, *η*^*2*^= 0], nor between respondents completing the survey on different devices [*F*(3, 296) = 0.402, *p *= 0.752, *η*^*2*^= 0.004]. However, there was a significant main effect of skincare regimen on utility magnitude [*F*(1.716, 507.801) = 42.675, *p *< 0.001, *η*^*2*^= 0.126]. Pairwise comparisons indicated that differences between skincare regimens likely to be followed by patients with good disease control (#4 to #7) did not differ significantly from one another (*p *> 0.1 in all cases), but among the skincare regimens for patients using TCS and emollient combinations (#1 to #3), there were some important differences. Specifically, the skincare regimen involving only a single application of TCS (#3) had statistically significantly higher utility ratings than the two regimens involving two applications of TCS (#1 and #2; *p *< 0.005 in both cases), which did not differ statistically from one another despite the difference in the number of emollient applications. Utility values for each of the skincare regimens likely to be followed by patients with good disease control (#4 to #7) were statistically significantly higher than any of those for patients using TCS and emollient combinations (#1 to #3; *p *< 0.001 in all cases).

### Disutilities

The largest disutilities were associated with use of TCS plus emollient skincare regimens (#1 to #3) vs those that are likely to be followed in patients with good disease control (#4 to #7; Table 4). Specifically, the two largest disutilities were between #1 (TCS twice daily and emollient four times daily) and #4 (light emollient twice daily) and #1 and #7 (light emollient on occasion, as needed); 0.1521 and 0.1705 respectively, indicating that the difference between the two treatment approaches would have the largest impact on patient QoL.

The disutilities between different skincare regimens that may be followed by patients with good disease control (#4 vs #5 and #5 vs #6) were negligible (0.0002 to 0.0049). On the other hand for skincare regimens that are followed by patients using TCS and emollient combinations, the impact of additional applications of emollient (#1 vs #2) had up to twice as large an impact on QoL than the impact of an additional application of TCS (#2 vs #3). For regimens involving two applications of emollient (#2, #3 and #4), there was an observable disutility of the addition of one application of TCS (#3 vs #4), which was greater still for two applications of TCS (#2 vs #4) compared with application of emollient only.

## Discussion

This study quantifies, for the first time, the societal value of the impact of topical AD treatments on QoL. Utility decreased with increases in the intensity and type of the skincare regimen. The most intense regimen (TCS twice daily and emollient four times daily) had a process utility of 0.7968, while the least intense regimen (light emollient application on occasion, as needed) was valued at 0.9999. No significant differences were observed between alternative skincare regimens that patients with good disease control may follow (0.9862 to 0.9999), however, when compared with skincare regimens involving TCS and emollient combinations (0.7968 to 0.8835), significant differences were demonstrated (*p* < 0.001 in all cases). Although just under half (44%) of respondents reported having used TCS to treat skin conditions, in line with evidence that more than 50% UK adults experience skin conditions within an average 12 month period [24] process utility ratings did not differ between those with and without previous TCS use.

Disutilities between the most and least intensive skincare regimens were of the greatest magnitude (− 0.1521). Disutilities between skincare regimens representative of patients with good disease control, were negligible (− 0.0039 to − 0.0049). Interestingly, respondents indicated twice as large an impact of the requirement for two additional applications of emollient cream (four applications per day vs. two; − 0.0436), compared with one additional application of TCS required per day (two applications per day vs. one; − 0.0214) in patients using the combination of TCS and emollient treatment. This suggests that for patients already following regimens including both TCS and emollient treatments, frequency and time spent applying applications may have a bigger impact on QoL than concern about perceived risk from more frequent use of TCS. However, the cumulative impact of increased required applications of TCS can be observed in the approximately 25% larger disutility observed when comparing disutilities between regimens #2 and #4 (− 0.1081), and #3 and #4 (− 0.0822). Regimens #2 and #3 differed only in the number of TCS applications, (two applications in regimen #2, one application in regimen #3) it is, therefore, likely that the larger disutility observed for the #2 vs. #4 compared with #3 vs. #4 reflects both increases in time required for application, as indicated in the skincare regimen descriptions, and negative perceptions around use of TCS.

The observed utility and disutility magnitude may be greater than anticipated, however the results show face validity, with respondents trading off little or no time for skincare regimens requiring minimal administration and time commitments, and increasing amounts of time for those with greater burden. A systematic review [21] of 15 studies valuing process utilities reported that the disutility of patch administration compared with oral administration was -0.16, similar in magnitude to the larger disutilities found in the current study, with a far higher disutility of − 0.27 observed for subcutaneous vs. oral administration. While reductions in dosage were only associated with relatively small utility gains between 0.01 and 0.09, none of the identified studies reported differences in daily topical treatment administration requirements.

A systematic review of published *health* utilities for the signs and symptoms of AD [25], identified values for moderate and severe disease severity in the range of 0.47 to 0.98, with the majority of values being in the region of 0.70. This is not far below the process utility obtained for the most intensive skincare regimen in the current study (0.80). Of course, there are inherent problems comparing utilities derived from different instruments, which may influence the magnitude of the values obtained. More importantly the two types of utility value (health and process) capture two different things; the former captures the impact of the signs and symptoms of a disease on QoL, which should be considered separately to the latter, which captures the impact of treatment processes. As such, one would expect that if both a ‘health utility’ and ‘process utility’ were captured in the same value, the magnitude would be a combination of the impact of both, resulting in a lower utility value still. This notion is supported by the fact that a value of 0.67 was elicited in patients using the SF-6D [26], which is unlikely to capture much disutility associated with treatment processes, while a lower value of 0.64 [27] was elicited with TTO vignettes that included brief descriptions of ‘daily use of emollients and topical anti-inflammatory treatments’, thus incorporating some aspects of both health and process. In order to truly measure the treatment benefit conferred by a new treatment, the utility metric should incorporate both. If aspects such as treatment process are to be included in metrics of treatment benefit used in economic evaluations, more research is needed to assess how ‘health’ and ‘process’ utility interact. It is likely that when considered in combination, the relationship between the two is more complex than simply resulting in an additive impact. For example, clinical benefit may reduce the disutility of burdensome processes in a metric that considers both health and process. Use of conjoint analysis techniques such as discrete choice experiments that include attributes concerning both disease control and treatment processes may be appropriate to more fully understand the relative value of each and the trade-offs patients are willing to make.

## Limitations

Although the Asian and British Asian, male, and over 65 year old respondent groups were slightly underrepresented compared with the targets specified, overall the sample recruited was large, and a good representation of the UK population with respect to age, gender and ethnicity. As such, the study provides estimates that are generalizable and likely accurately reflect the societal preferences of the UK, in line with the NICE reference case [28].

Floor effects were observed for the skincare regimens reflecting patients with good disease control. Such effects are consistent with the bounding of utility values at 1 in utility elicitation, and, while some researchers have proposed use of statistical or procedural methods to address these (such as censored regression techniques or valuation of states that exceed 1 [29]), this is still somewhat controversial and can be difficult to implement. Consequently, exclusion of outliers resulted in a smaller sample for these health states, and left very little room for variability, particularly for skincare regimens 6 and 7. However, given that the skincare regimens described in these items were of such low intensity, and are typically followed by many individuals in the general population without AD, we feel that the results were plausible. It should be noted that, in general, disutilities calculated at the respondent level (i.e. including only respondents who provided a non-outlying response to both items required to calculate the disutility) were smaller than those calculated at the grand mean level (i.e. the difference between the mean values reported in Table 3), indicating that these values are more conservative. Alternative methods of exclusion were evaluated in sensitivity analyses (see Supplement A) to assess whether they may have improved any possible bias from the statistical outlier approach. Some of these analyses resulted in paradoxical positive disutility scores between the good disease control skincare regimens, indicating that while they may have provided more conservative estimates of disutility, the alternative methods may also have retained some respondents with inconsistent and theoretically implausible responses.

Electronic administration allows fast collection of data from large samples, but some nuances and opportunities for respondents to reflect that would be possible in an interview setting, are lost. The current study benefits from realistic vignettes developed rigorously on the basis of literature and with input from healthcare professionals specialising in AD care; however, ‘vignette’ studies are limited. When asked to imagine that they have an illness, or, in this case, follow a skincare regimen, respondents typically imagine the impact at the time immediately following diagnosis and respond to questions accordingly. In contrast, patients often grow accustomed to living with a condition and although their perceived health utility may be lower at disease onset, it commonly improves as patients get used to the condition [30]. In the case of topical treatment processes, the generally poor adherence to skincare regimens, which is observed in clinical practice [31, 32], suggests that AD patients may not adapt to their recommended skincare regimens in the same way patients may learn to cope with the signs and symptoms of a disease. This study may also have detected negative perceptions around the use of TCS as well as the impact of treatment process on quality of life. However, for patients, the detrimental impacts of treatment processes are balanced against the beneficial clinical effect, and this has not been examined in the current study.

While the TTO approach is well documented and recommended in NICE guidelines, task engagement and comprehension can limit data quality. There was a large range in the time that some respondents were willing to trade off, with some individuals trading in the full 10 years of life for a treatment which would, therefore, mean immediate death. It is likely that, for some respondents, there was some confusion about the nature of the task and the ratings that they were making, and that others were simply completing the task as quickly as possible and not giving the necessary consideration to the task at hand. Despite concerns, average completion time was 9.9 min, which aligned with the survey design target for a 10-min completion. Randomisation of the order of health state presentation across participants aimed to reduce order effects and exclusion of outliers is likely to have removed a large proportion of unthoughtful or confused respondents.

The TTO procedure implemented in this study used only a single, relatively short, time frame of 10 years, the choice of which may have influenced the magnitude of the utilities observed. The 10-year time frame has been used extensively, including in the TTO valuation of EQ-5D health states, the preferred methodology for health utility elicitation by NICE. In this case, both to align with NICE methodology, and to minimise difficulties for respondents in considering the impact of treatment processes beyond a point in the future where their lifestyle may have altered considerably (e.g. retirement, old age), the 10-year time frame was considered most appropriate. Although there is evidence of a tendency for shorter time frames to result in lower utility values, the relationship between time frame and magnitude is not consistent across studies [33]; thus it is unclear how time frame may have impacted valuation. Similarly, rather than the traditional iterative elicitation technique, this study requested direct statements of the maximum length of time respondents were willing to trade off. While this approach limits the risk of some of the biases likely to impact iterative approaches [34], it remains unknown whether it introduces other biases, and which direction these may fall in.

## Conclusions

To our knowledge, this is the first study which quantifies the detrimental impact on QoL due to topical treatment for patients with AD and shows that this increases with time spent and number of applications. The magnitude of these values highlights the importance the UK general public place on the time required for topical applications, unpleasant cosmetic properties and interference with daily life that are associated with intensive skincare regimens. Moreover, it further emphasises likely reasons behind poor treatment adherence and the need for better patient support and optimal treatments that provide clinical relief and can be easily integrated into daily life.

As the first study to quantify the impact of the treatment processes on QoL, previously documented only qualitatively, these findings add to the sparse literature on ‘process utility’. Quantification of process utility is the first step towards facilitating integration of non-health treatment benefits into the quantitative evidence considered by decision-makers. While the nature of the relationship between health and process utilities remains unclear, our findings effectively demonstrate the magnitude of the impact treatment processes may have in AD, and suggests that this element of treatment should not be considered trivial or neglected in the decision-making process.

## Electronic supplementary material

Below is the link to the electronic supplementary material.

Supplementary material 1 (DOCX 42 kb)

Supplementary material 2 (DOCX 52 kb)
