# Supplementary material for: Process utilities for topical treatment in atopic dermatitis
Source: Qual Life Res. 2019 Apr 3;28(9):2373–81. doi: 10.1007/s11136-019-02174-0 (PMC6698259; doi:10.1007/s11136-019-02174-0)
Supplement: Supplementary file 1 — Supplementary material 1 (DOCX 42 kb) [file 11136_2019_2174_MOESM1_ESM.docx]

**Supplement A: Sensitivity of Outlier Removal**

In order to assess uncertainty around the values, and to address potential bias resulting from the use of the interquartile range-based rule for exclusion where floor effects were present, a series of sensitivity analyses were conducted. Alternative exclusion criteria were developed and utility and disutility values were recalculated. Specifically, the impact of the following different exclusion criteria was assessed:

- No exclusions;
- Strict exclusion, whereby any respondent with an invalid response (according to the IQR criteria) for **any** skincare regimen was excluded from **all** calculations;
- Commonly invalid exclusion, whereby any respondent with an invalid response (according to the IQR criteria) for **all** of skincare regimens 4, 5, 6 and 7 (those for which floor effects were observed), was excluded from **all** calculations;
- Rules applied, whereby any respondent who responded to **any** skincare regimen with a 10 year trade-off, **and/or** who did not trade-off a greater number of years for regimen 1 than 7 (with the exception of people who responded with a 0 for both states), thereby indicating poor understanding of either the task itself, or of the response scale, was excluded.

Utility and disutility values calculated using these rules are listed in Tables S1 and S2 respectively.

**Table S1: Mean (SD) utilities for the base case and sensitivity analyses**

| **No.** | **Skincare regimen** | **Base case**  ***n*=variable ^a^** | **No exclusions**  ***n*=484** | **Strict exclusion**  ***n*=304** | **Commonly invalid**  ***n*=438** | **Rules applied**  ***n*=418** |
| --- | --- | --- | --- | --- | --- | --- |
| **1** | Steroid twice daily and emollient four times daily | .7968  (.2159) | .7793  (.2426) | .8518  (.1672) | .8062  (.2248) | .7899  (.2192) |
| **2** | Steroid twice daily and emollient twice daily | .8471  (.1744) | .8205  (.2189) | .8993  (.1318) | .8511  (.1908) | .8467  (.1808) |
| **3** | Steroid once daily and emollient twice daily | .8835  (.1469) | .8337  (.2251) | .9143  (1209) | .8615  (.1964) | .8608  (.1887) |
| **4** | Light emollient twice daily | .9862  (.0340) | .9149  (.1867) | .9945  (.0218) | .9560  (.1252) | .9446  (.1347) |
| **5** | Light emollient once daily | .9906  (.0267) | .9098  (.1990) | .9964  (.0168) | .9526  (.1252) | .9425  (.1514) |
| **6** | Light emollient once every other day | .9997 (  .0021) | .9249  (.1878) | .9999  (.0008) | .9659  (.1198) | .9546  (.1441) |
| **7** | Light emollient on occasion, as needed | .9999  (.0012) | .9213  (.1888) | .9999  (.0011) | .9619  (.1263) | .9656  (.1155) |

^a^ To see numbers of respondents included in each comparison, please refer to Table 3 in the main text.

Figure S1 indicates that, overall, although the magnitude of the utility values was different across the sensitivity analyses, the pattern of results was the same regardless of the exclusion rule applied, with utility increasing with decreases in the intensity of the skincare regimen. The analysis involving no exclusions generated the lowest utility values, while that with the strict exclusion based on the IQR criteria generated the highest utility values. These measures reflect the lower and upper limits of these values, separated by a magnitude of around 0.075. This is in line with expectations, given that outliers identified by the IQR criteria were those who had traded-off atypically high periods of time to avoid following the given skincare regimens (thus producing lower utility for that regimen) compared with the general pattern of results. As such, inclusion of all respondents, including those who produced very low utilities, led to a reduction the mean, while strict exclusion based on this rule led to exclusion of a greater number of respondents with low utility, leading to a higher mean.

**Figure S1: Mean utility values generated in the sensitivity analyses**

Interestingly, estimates generated through rule-based exclusion and exclusion of respondents commonly giving invalid responses (according to the IQR criteria) were similar for all skincare regimens, with those generated through rule-based exclusion being slightly lower in general. The base case estimates were similar to these for skincare regimens involving steroid and emollient, but for those regimens reflecting possible scenarios for patients with good disease control, utilities produced by the base case analysis were higher, and more similar to those produced using the strict exclusion criteria.

**Table S2: Mean disutilities for the base case and sensitivity analyses**

| **Nos.** | **Change in skincare regimen** | **Base case**  ***n*=variable ^a^** | | **No exclusions**  ***n*=484** | **Strict exclusion**  ***n*=304** | **Commonly invalid**  ***n*=438** | **Rules applied**  ***n*=418** |
| --- | --- | --- | --- | --- | --- | --- | --- |
|  |  | Respondent level | Grand mean level |  |  |  |  |
| **1 vs 4** | Steroid + emollient as advised vs good disease control as advised | -.1521 | -.1894 | -.1356 | -.1427 | -.1498 | -.1547 |
| **1 vs 7** | Steroid + emollient as advised vs best case scenario with very good disease control | -.1705 | -.2031 | -.1420 | -.1481 | -.1557 | -.1757 |
| **1 vs 2** | 2 additional applications of emollient in patients using steroid + emollient | -.0436 | -.0503 | -.0412 | -.0475 | -.0449 | -.0568 |
| **2 vs 3** | 1 additional application of steroid in patients using steroid + emollient | -.0214 | -.0364 | -.0132 | -.0150 | -.0104 | -.0141 |
| **2 vs 4** | 2 applications of steroid, plus heavier emollient in patients applying 2 applications of emollient | -.1081 | -.1391 | -.0944 | -.0952 | -.1049 | -.0979 |
| **3 vs 4** | 1 application of steroid, plus heavier emollient in patients applying 2 applications of emollient | -.0822 | -.1027 | -.0812 | -.0802 | -.0945 | -.0838 |
| **4 vs 5** | 1 additional application of light emollient in patients with good disease control | -.0039 | -.0044 | .0051 | -.0019 | .0034 | .0021 |
| **5 vs 6** | Daily vs. bi-daily application of light emollient in patients with good disease control | -.0049 | -.0002 | .0036 | .0000 | .0040 | -.0110 |

^a^ To see numbers of respondents included in each comparison, please refer to Table 4 in the main text.. Please note that for all sensitivity analyses the number of respondents was the same across all skincare regimens, therefore the disutilities calculated at the respondent-level would be the same as those calculated at the grand mean level.

Figure S2 shows that, as with the utility values, the broad pattern of results was similar across the disutilities produced by the sensitivity analyses. Calculations using the grand means in base case analysis most often produced the disutility values of greatest magnitude, while those produced at the respondent level were more conservative, and those from the analysis with no exclusions produced those of the smallest magnitude. Disutilities between regimens 4 and 5, and 5 and 6 were negligible in all analyses, however the differences between these health states for some of the sensitivity analyses (commonly invalid, rules applied, no exclusion) were in an unexpected direction. This likely reflects the fact that these skincare regimens described such similarly small impacts on the HRQoL of patients, that respondents found it difficult to differentiate between, and may indicate that these analyses continued to include respondents who provided inconsistent responses.

**Figure S2: Mean disutility values generated in the sensitivity analyses**

Table S3 summarises the sample characteristics for the subsets of respondents included in each sensitivity analysis. It can be seen that despite different total numbers of respondents included in each subset, the sample characteristics remained very similar, differing by an average of only 1.6% (SD: 1.1%) between the subset with the largest proportion of respondents within a particular category and that with the smallest proportion. This suggests that exclusions did not systematically affect particular groups of respondents based on the characteristics measured.

**Table S3: Sample characteristics for sensitivity analyses**

| **Characteristic** | **No exclusions**  ***n*=484** | **Strict exclusion**  ***n*=304** | **Commonly invalid**  ***n*=438** | **Rules applied**  ***n*=418** |
| --- | --- | --- | --- | --- |
| **Gender** |  |  |  |  |
| Male | 229 (47.3%) | 131 (43.1%) | 205 (46.8%) | 197 (47.1%) |
| Female | 255 (52.7%) | 173 (56.9%) | 233 (53.2%) | 221 (52.9%) |
| **Age** |  |  |  |  |
| 18-24 | 55 (11.4%) | 26 (8.6%) | 49 (11.2%) | 44 (10.5%) |
| 25-34 | 85 (17.6%) | 48 (15.8%) | 73 (16.7%) | 69 (16.5%) |
| 35-44 | 80 (16.5%) | 51 (16.8%) | 68 (15.5%) | 70 (16.7%) |
| 45-54 | 90 (18.6%) | 62 (20.4%) | 84 (19.2%) | 82 (19.6%) |
| 55-64 | 70 (14.5%) | 48 (15.8%) | 67 (15.3%) | 61 (14.6%) |
| 65 and over | 104 (21.5%) | 69 (22.7%) | 97 (22.1%) | 92 (22.0%) |
| **Ethnicity** |  |  |  |  |
| White or White British | 435 (89.9%) | 275 (90.5%) | 396 (90.4%) | 379 (90.7%) |
| Asian or Asian British | 22 (4.5%) | 12 (3.9%) | 17 (3.9%) | 17 (4.1%) |
| Black or Black British | 13 (2.7%) | 9 (3.0%) | 11 (2.5%) | 11 (2.6%) |
| Mixed or Multiple | 9 (1.9%) | 5 (1.6%) | 9 (2.1%) | 6 (1.4%) |
| Other | 5 (1.0%) | 3 (1.0%) | 5 (1.1%) | 5 (1.2%) |
| **Previous use of steroid creams** |  |  |  |  |
| Yes | 214 (44.2%) | 134 (44.1%) | 186 (42.5%) | 181 (43.3%) |
| No | 270 (55.8%) | 170 (55.9%) | 252 (57.5%) | 237 (56.7%) |
| **Country of habitation** |  |  |  |  |
| England | 413 (85.3%) | 262 (86.2%) | 377 (86.1%) | 355 (84.9%) |
| Scotland | 40 (9.3%) | 25 (8.2%) | 35 (8.0%) | 37 (8.9%) |
| Wales | 24 (5.0%) | 12 (3.9%) | 19 (4.3%) | 20 (4.8%) |
| Northern Ireland | 7 (1.4%) | 5 (1.6%) | 7 (1.6%) | 6 (1.4%) |
| **Device** |  |  |  |  |
| Smartphone | 14 (2.9%) | 5 (1.6%) | 12 (2.7%) | 11 (2.6%) |
| Laptop | 250 (51.7%) | 159 (52.3%) | 224 (51.1%) | 208 (49.8%) |
| Computer | 137 (28.3%) | 77 (25.3%) | 124 (28.2%) | 122 (29.2%) |
| Tablet | 83 (17.1%) | 63 (20.7%) | 78 (17.8%) | 77 (18.4%) |
