# Supplementary material for: Process utilities for topical treatment in atopic dermatitis
Source: Qual Life Res. 2019 Apr 3;28(9):2373–81. doi: 10.1007/s11136-019-02174-0 (PMC6698259; doi:10.1007/s11136-019-02174-0)
Supplement: Supplementary file 2 — Supplementary material 2 (DOCX 52 kb) [file 11136_2019_2174_MOESM2_ESM.docx]

You are invited take part in a study being run by York Health Economics Consortium (YHEC) and sponsored by a pharmaceutical company. We would like to know about how much you value the impact that different skincare routines for severe skin conditions have on quality of life.

This survey has 2 parts to it. The first part of the survey asks you some questions about yourself. These questions do not ask for any personal information, and you will not be asked to provide your name, address or date of birth, or any other information which could be linked to you.

For the second part, there will be 7 two-part questions in which we would like you to imagine you are following the skincare routine we have described to control symptoms of a severe skin condition.

Please **only** complete this on a **computer, laptop or tablet device**. The questions will not be presented accurately on a smartphone. If you are on a smartphone, do **not** complete the survey now, please wait until you are at a computer before proceeding further.

*<< Next page >>*

What is your age?

- 18-24
- 25-34
- 35-44
- 45-54
- 55-64
- 65 or over

What is your gender?

- Male
- Female

Which of these countries do you live in?

- England
- Wales
- Northern Ireland
- Scotland
- None of the above

What is your ethnicity?

- White or White British
- Asian or Asian British
- Black or Black British
- Mixed or Multiple
- Other

Do you currently, or have you ever, used steroid-based creams or ointments to treat skin conditions?

- Yes
- No

Please select the device below that best describes the device you are using to complete this survey.

- Smartphone
- Laptop
- Tablet
- Computer
- Other

*<< Next page. If ‘none of the above’ is selected in response to country of habitation, then the survey is terminated instead of proceeding to the next page. If ‘smartphone’ is selected as the device, then the following is presented, but respondents can still proceed>>*

Remember, these questions will not show up well on a smartphone. Please return to this survey when you are at a computer, laptop or tablet.

*<< Next page >>*

Atopic dermatitis is the most severe type of eczema. It is a skin disease that causes persistent and widespread redness, rashes, and scaly patches. Patients say that the worst symptom is the itching, which can make it difficult to concentrate and to sleep at night.

The most common way to treat atopic dermatitis is to use two different products together. The first is a steroid-based product (usually a cream or an ointment) that patients apply to the inflamed areas of skin. The second is an emollient product (usually a cream or ointment) that patients apply to their whole body to keep skin hydrated. These products need to be applied multiple times a day to control the symptoms of atopic dermatitis.

Applying the products is time consuming. It can take up to an hour from the application of the first product before the second product has been fully absorbed. Only then can patients get dressed. This can be difficult to fit into a patient’s personal and working life. Finding a place where it is possible to undress, apply the products, and wait for them to be absorbed, can be difficult when out of the house (e.g. at work). If patients have plans to do anything outside their normal routine, they need to consider when and where they will be able to apply their treatment products.

The products are often greasy, so they can feel uncomfortable, look shiny, and transfer onto things. They transfer onto clothing and make it stick to the skin, so patients need to be careful what type of clothes they buy and wash them more regularly. Patients often need to reapply emollient products to their hands throughout the day as hand-washing can dry out their skin. This may cause problems with greasy transfer onto paper and work documents and computer keyboards, which may be inconvenient and embarrassing. Greasy hands may also be a safety hazard when transfer makes surfaces slippery e.g. for driving or handling machinery. Patients may be concerned about repeated use of steroid products if they think that it can make the skin more fragile and damage their immune system. Patients may also have lowered self-esteem when using these products. They may not wish for colleagues to know about their condition and have to think of ways to conceal reapplications. They may find it difficult to build new relationships and explain their treatment to new partners. Patients may also be restricted in the sports they feel happy to take part in.

*<< Next page >>*

In the next part of the survey we would like you to imagine you are a patient who has atopic dermatitis, and that you have 10 years left to live.

In each question, we will describe a different skincare routine we want you to imagine you have to follow to control your symptoms, and describe how this might affect your daily life. We will then ask you to decide whether you think it would be worth it to give up some of your remaining 10 years of life so that you no longer have to follow the skincare routine described.

If you think that it would be worth it, we will ask you to move a slider to show what the maximum amount of time would be that you would give up. We will show the description of the skincare routine on the page so that you can look back at it while you consider your answer.

For example, you might think we have described a skincare routine that you would find very hard to live with. You might think it would be worth 3 years of your life so that you would not have to follow the routine every day, even if it meant you could live only 7 years instead of 10. To show this, you would move the slider like this:

*
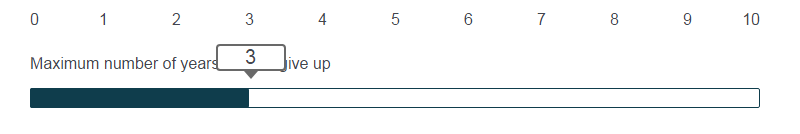
*

This would mean that if you are 30 years old now, you would be choosing to live to the age of 37 without having to apply creams, instead of living to the age of 40 but having to follow the described skincare routine.

You can choose to give up less than a year if you think that the skincare routine described would be easy enough to live with, but that avoiding the inconvenience would be worth a few months of life. For example, you could decide to give up 6 months (or 0.5 years).

You do not have to give up any of your life, but if you choose 0 years, that is the same as saying that you would be unwilling to live for a shorter time. That would mean you would be choosing to live the full 10 years and would have to follow the skincare routine for all of those 10 years.

*<< Next page. The following 7 two-part items will be presented to respondents in a randomized order. >>*

**Now imagine that:**

*Skincare routine:*

- You need to apply steroid product to the inflamed areas of skin **twice each day**.
- You need to apply an emollient product to your whole body **four times each day**.
- This means **two applications** of steroid plus emollient **that take 55 minutes each** ( 20 minutes for application and 35 minutes drying time).
- And, **in addition, two applications** of emollient only **that take 25 minutes each** (5 minutes application, 20 minutes drying time).
- That’s a total of **4 applications** taking **2 hours and 40 minutes** altogether.

*How it might impact your daily life:*

- You may find it very hard to fit four applications, including two long applications, into your work and personal life.
- You have to reapply these products throughout the day, including when you are out of the house (e.g. at work, shopping). This means finding a place to undress, reapply, and wait for the product to be absorbed. This place could be a public bathroom.
- The products you use transfer onto your clothes, and affect your choice of clothes on a daily basis.
- You need to reapply the products to your hands throughout the day, every day, and these can transfer to anything you touch.
- You need to plan carefully how you will continue your skincare routine for an evening out, a day out, or an overnight stay, as well as longer holidays or business trips.
- You are restricted in the sports you can do on a daily basis.
- You may be worried about the impact of using steroid products so often.
- You may feel self-conscious about how the products make your skin shiny and the distinctive ‘medicinal’ smell on a daily basis.
- You may need to explain your skincare routine to colleagues, new friends and new partners. This may be embarrassing.

Imagine you have 10 years left to live, and in those 10 years you will have to follow the skincare routine described above.

Would you be willing to live for a shorter time (i.e. trade-in some of your remaining life) so that you no longer need to follow the skincare routine described above?

- Yes
- No

*<< Next page. If ‘yes’ was selected, respondents will then see the following screen for the second part of this item. If ‘no’ was selected, respondents will skip to the next item. >>*

Out of the 10 years you have left to live, what is the **maximum** length of time you would consider it worthwhile to give up so that you do not need to follow the skincare routine described (shown again below as a reminder)?

- **Two applications, every day,** of steroid plus emollient that **take 55 minutes each.**
- Plus**, two applications, every day,** of emollient only, **that take 25 minutes each**.


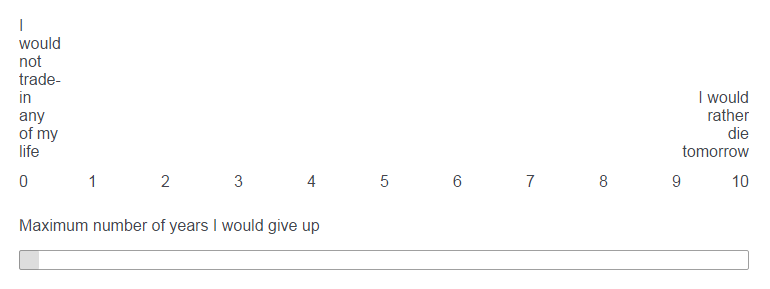


Please note, an answer of 0 indicates that you would **not** be willing to trade-in any of your remaining life, therefore you would live for the full 10 years and would have to follow the skincare routine.

*<< Next page >>*

**Now imagine that:**

*Skincare routine:*

- You need to apply steroid cream to the inflamed areas of skin **twice each day**.
- You need to apply an emollient product to your whole body **twice each day**.
- This means **two applications** of steroid plus emollient, **that take 55 minutes each** (20 minutes for application and 35 minutes drying time).
- That’s a total of **2 applications** taking **1 hour and 50 minutes** altogether, **every day**.

*How this might impact your daily life:*

- You may find it hard to fit two long applications into your work and personal life.
- The products you use transfer onto your clothes, and affect your choice of clothes on a daily basis.
- You need to reapply the products to your hands throughout the day, every day, and these can transfer to anything you touch.
- You need to plan carefully how you will continue your skincare routine for an evening out, a day out, or an overnight stay, as well as longer holidays or business trips.
- You are restricted in the sports you can do on a daily basis.
- You may be worried about the impact of using steroid products so often
- You may feel self-conscious about how the products make your skin shiny and the distinctive ‘medicinal’ smell on a daily basis.
- You may need to explain your skincare routine to colleagues, new friends and new partners. This may be embarrassing.

Imagine you have 10 years left to live, and in those 10 years you will have to follow the skincare routine described above.

Would you be willing to live for a shorter time (i.e. trade-in some of your remaining life) in order to be cured so that you no longer need to follow the skincare routine described above?

- Yes
- No

*<< Next page. If ‘yes’ was selected, respondents will then see the following screen for the second part of this item. If ‘no’ was selected, respondents will skip to the next item. >>*

Out of the 10 years you have left to live, what is the **maximum** length of time you would consider it worthwhile to give up so that you do not need to follow the skincare routine described (shown again below as a reminder)?

- **Two applications, every day,** of steroid plus emollient that **take 55 minutes each**.


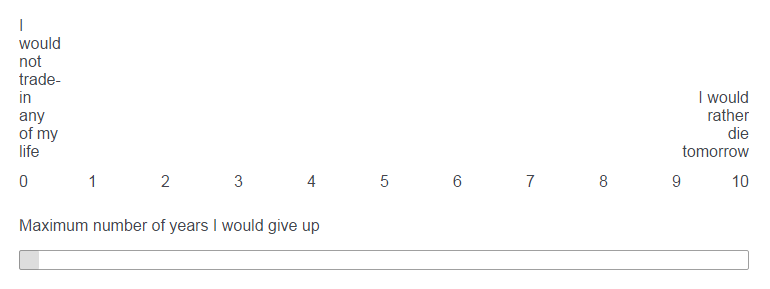


Please note, an answer of 0 indicates that you would **not** be willing to trade-in any of your remaining life, therefore you would live for the full 10 years and would have to follow the skincare routine.

*<< Next page >>*

**Now imagine that:**

*Skincare routine:*

- You need to apply steroid cream to the inflamed areas of skin **once each day**.
- You need to apply an emollient product to your whole body **twice each day**.
- This means **one application** of steroid plus emollient, **which takes** **55 minutes** (20 minutes for application and 35 minutes drying time).
- And, **in addition, one application** of emollient only, **which takes 25 minutes** (5 minutes application, 20 minutes drying time).
- That’s a total of **2 applications** taking **1 hour and 20 minutes** altogether, **every day**.

*How it might impact your daily life:*

- You find it hard to fit both applications, including one long application, into your work and personal life.
- The products you use transfer onto your clothes, and affect your choice of clothes on a daily basis.
- You need to reapply the products to your hands throughout the day, every day, and these can transfer to anything you touch.
- You need to plan carefully how you will continue your skincare routine for an evening out, or an overnight stay, as well as longer holidays or business trips.
- You are restricted in the sports you can do on a daily basis.
- You may be worried about the impact of using steroid products so often.
- You may self-conscious about how the products make your skin shiny and the distinctive ‘medicinal’ smell on a daily basis.
- You may need to explain your skincare routine to colleagues, new friends and new partners. This may be embarrassing.

Imagine you have 10 years left to live, and in those 10 years you will have to follow the skincare routine described above.

Would you be willing to live for a shorter time (i.e. trade-in some of your remaining life) in order to be cured so that you no longer need to follow the skincare routine described above?

- Yes
- No

*<< Next page. If ‘yes’ was selected, respondents will then see the following screen for the second part of this item. If ‘no’ was selected, respondents will skip to the next item. >>*

Out of the 10 years you have left to live, what is the **maximum** length of time you would consider it worthwhile to give up so that you do not need to follow the skincare routine described (shown again below as a reminder)?

- **One application, every day,** of steroid plus emollient, **which takes** **55 minutes**.
- **Plus, one application, every day,** of emollient only, **which takes 25 minutes**.


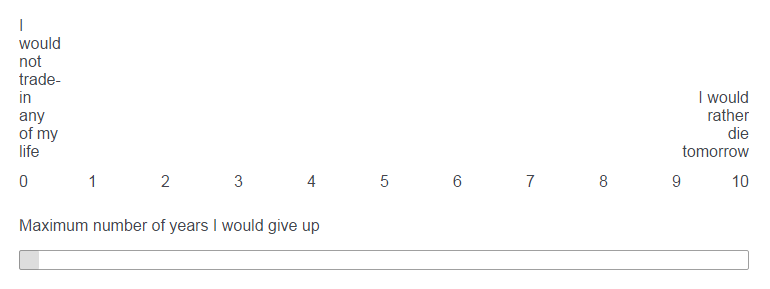


Please note, an answer of 0 indicates that you would **not** be willing to trade-in any of your remaining life, therefore you would live for the full 10 years and would have to follow the skincare routine.

*<< Next page >>*

**Now imagine that:**

*Skincare routine:*

- You need to apply a **light** emollient product to your whole body **twice each day**.
- This means **two applications** of emollient only, **which take 15 minutes each** (5 minutes application, 10 minutes drying time).
- That’s a total of **2 applications** taking **30 minutes** altogether **every day**.

*How it might impact your daily life:*

- You may find it annoying to fit both applications into your work and personal life.
- You need to reapply the product to your hands throughout the day, on a daily basis.
- You need to plan carefully how you will continue your skincare routine for an overnight stay, a longer holiday or business trip.
- You may feel self-conscious about the product’s distinctive ‘medicinal’ smell on a daily basis.
- You may need to explain your skincare routine to colleagues, new friends and new partners. This may be embarrassing.

Imagine you have 10 years left to live, and in those 10 years you will have to follow the skincare routine described above.

Would you be willing to live for a shorter time (i.e. trade-in some of your remaining life) in order to be cured so that you no longer need to follow the skincare routine described above?

- Yes
- No

*<< Next page. If ‘yes’ was selected, respondents will then see the following screen for the second part of this item. If ‘no’ was selected, respondents will skip to the next item. >>*

Out of the 10 years you have left to live, what is the **maximum** length of time you would consider it worthwhile to give up so that you do not need to follow the skincare routine described (shown again below as a reminder)?

- **Two applications,** **every day,** of **light** emollient only, **which take 15 minutes each**.


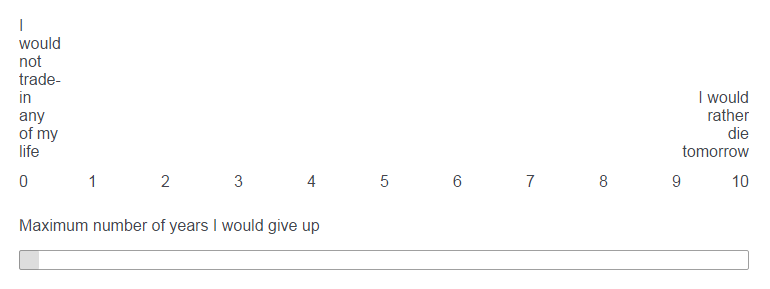


Please note, an answer of 0 indicates that you would **not** be willing to trade-in any of your remaining life, therefore you would live for the full 10 years and would have to follow the skincare routine.

*<< Next page >>*

**Now imagine that:**

*Skincare routine:*

- You need to apply a **light** emollient product to your whole body **once each day**.
- This means **one application** taking a total of **15 minutes, every day** (5 minutes application, 10 minutes drying time).

*How it might impact your daily life:*

- You need to reapply the product to your hands throughout the day, on a daily basis.
- You need to plan carefully how you will continue your skincare routine for an overnight stay, a longer holiday or business trip.
- You may feel self-conscious about the product’s distinctive ‘medicinal’ smell on a daily basis.
- You may need to explain your skincare routine to colleagues, new friends and new partners. This may be embarrassing.

Imagine you have 10 years left to live, and in those 10 years you will have to follow the skincare routine described above.

Would you be willing to live for a shorter time (i.e. trade-in some of your remaining life) in order to be cured so that you no longer need to follow the skincare routine described above?

- Yes
- No

*<< Next page. If ‘yes’ was selected, respondents will then see the following screen for the second part of this item. If ‘no’ was selected, respondents will skip to the next item. >>*

Out of the 10 years you have left to live, what is the **maximum** length of time you would consider it worthwhile to give up so that you do not need to follow the skincare routine described (shown again below as a reminder)?

- **One application,** **every day,** of **light** emollient only, **which takes 15 minutes**.


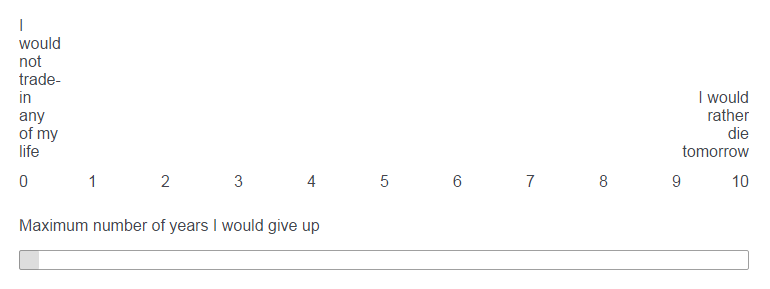


Please note, an answer of 0 indicates that you would **not** be willing to trade-in any of your remaining life, therefore you would live for the full 10 years and would have to follow the skincare routine.

*<< Next page >>*

**Now imagine that:**

*Skincare routine:*

- You need to apply a **light** emollient product to your whole body **once every other day**.
- This means **one application** taking a total of **15 minutes, every day** (5 minutes application, 10 minutes drying time).

*How it might impact your daily life:*

- You need to plan carefully how you will continue your skincare routine for a longer holiday (2+ nights) or business trip.
- You may feel self-conscious about the product’s distinctive ‘medicinal’ smell on days where you use it.

Imagine you have 10 years left to live, and in those 10 years you will have to follow the skincare routine described above.

Would you be willing to live for a shorter time (i.e. trade-in some of your remaining life) in order to be cured so that you no longer need to follow the skincare routine described above?

- Yes
- No

*<< Next page. If ‘yes’ was selected, respondents will then see the following screen for the second part of this item. If ‘no’ was selected, respondents will skip to the next item. >>*

Out of the 10 years you have left to live, what is the **maximum** length of time you would consider it worthwhile to give up so that you do not need to follow the skincare routine described (shown again below as a reminder)?

- **One application,** **every other day,** of **light** emollient only, **which takes 15 minutes**.


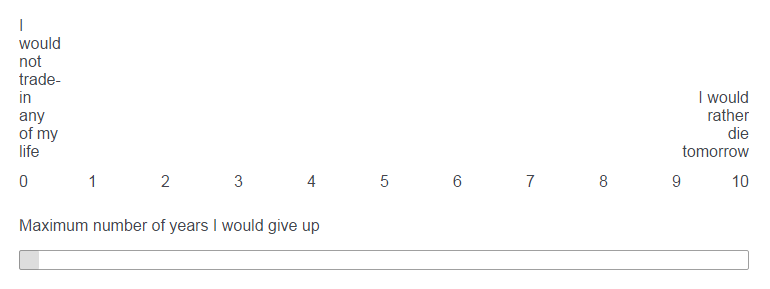


Please note, an answer of 0 indicates that you would **not** be willing to trade-in any of your remaining life, therefore you would live for the full 10 years and would have to follow the skincare routine.

*<< Next page >>*

**Now imagine that:**

*Skincare routine:*

- **On occasion**, when your skin feels dry or itchy, you need to apply a **light** emollient product to your whole body **once**.
- This means **one application** taking a total of **15 minutes, on occasion** (5 minutes application, 10 minutes drying time).

*How it might impact your daily life:*

- On these days you need to plan extra time into your morning or evening routine to fit this application in.
- On these days you may feel self-conscious about the product’s distinctive ‘medicinal’ smell.

Imagine you have 10 years left to live, and in those 10 years you will have to follow the skincare routine described above.

Would you be willing to live for a shorter time (i.e. trade-in some of your remaining life) in order to be cured so that you no longer need to follow the skincare routine described above?

- Yes
- No

*<< Next page. If ‘yes’ was selected, respondents will then see the following screen for the second part of this item. If ‘no’ was selected, respondents will skip to the next item. >>*

Out of the 10 years you have left to live, what is the **maximum** length of time you would consider it worthwhile to give up so that you do not need to follow the skincare routine described (shown again below as a reminder)?

- **One application,** **occasionally,** of **light** emollient only, **which takes 15 minutes**.


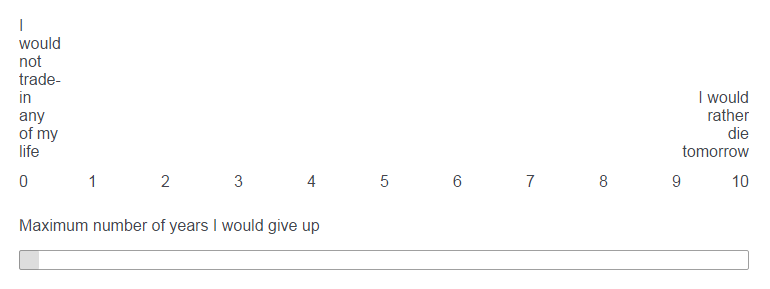


Please note, an answer of 0 indicates that you would **not** be willing to trade-in any of your remaining life, therefore you would live for the full 10 years and would have to follow the skincare routine.

*<< End of survey >>*
